# Behavioral interactions and emotional engagement with loss-related objects in bereavement: associations with grief symptom severity

**DOI:** 10.1186/s40359-026-04784-z

**Published:** 2026-05-21

**Authors:** Gizem Cesur Soysal, Holly G. Prigerson, Richard D. Goldstein, Ela Arı, Emrah Keser

**Affiliations:** 1 Department of Psychology, Istanbul Medipol University, Istanbul, Turkey; 2 Center for Research On End-of-Life Care, Weill Cornell Medicine, New York City, NY USA; 3 Division of General Pediatrics, Department of Pediatrics, Boston Children’s Hospital and Harvard Medical School, Boston, USA; 4 Department of Psychology, TED University, Ankara, Turkey

**Keywords:** Grief Symptoms, Loss-Related Objects, Attachment, Emotion Regulation

## Abstract

**Background:**

Grief involves attachment- related regulatory processes through which individuals adapt to separation from the deceased. Loss-related objects (i.e., emotionally significant belongings of the deceased) may reflect may reflect externalized expressions of proximity-seeking, yet their association with grief symptoms remains understudied.

**Aims:**

This study examines the role of loss-related objects in the grieving process by investigating their associations with attachment styles, difficulties in emotion regulation, and grief symptom severity.

**Method:**

The sample consisted of 250 adults who had experienced the death of a loved one. Participants completed the Experiences in Close Relationships-Revised Short Form, the Difficulties in Emotion Regulation Scale-Brief Form, loss object related items, and the Prolonged Grief Scale-13 (PG-13).

**Results:**

Findings revealed that 83.2% of participants kept a loss-related object. Those who kept objects exhibited higher grief symptom scores (*t* = 3.36, *p* = .001) and impulse control difficulties (*t* = 2.07, *p* = .009), whereas non-keepers showed higher avoidant attachment (*t* = -2.02, *p* = .043). PG-13 scores was associated with shorter time since loss (*β* = -.16), higher avoidant attachment (*β* = .13), greater difficulties in emotion regulation strategies (*β* = .44), and object-related interactions, including visiting frequency (*β* = .14), smelling the object (*β* = .21), and pain experienced during interactions with the object (*β* = .27).

**Conclusions:**

Loss-related objects may function as externalized representations within the grief process. Engagement with these objects was associated with grief severity beyond attachment and emotion regulation, suggesting that assessing such interactions may help identify risk and inform intervention targets.

**Supplementary Information:**

The online version contains supplementary material available at 10.1186/s40359-026-04784-z.

## Introduction

The death of a loved one can be one of the most devastating experiences a person may face. Death results in an irreversible physical separation from a loved one and makes their return impossible. Bereaved survivors are placed in a position of needing to adjust to this separation, a process referred to as grief. While grief is a normal, natural process, it can sometimes become profoundly distressing and drain vitality, leaving lasting effects on psychological well-being, occupational functioning, and interpersonal relationships. This condition, known as Prolonged Grief Disorder (PGD), is characterized by symptoms such as separation distress, intense emotional pain, identity disruption, disbelief about the death, avoidance, emotional numbness, loneliness, and feelings of meaninglessness or emptiness, persisting for more than a year and impairing social or occupational functioning [1, 2].

According to attachment theory, relationships with loved ones play a crucial role in emotion regulation [3]. Early in life, caregivers provide a secure base that enables exploration and emotion regulation; over time, this source of security becomes internalized, allowing individuals to regulate emotions independently [4, 5]. Following the death of a loved one, the attachment system is activated, often giving rise to an initial search for the deceased’s physical presence—such as visiting the deceased's grave, speaking to them, or using their belongings. This reflects a mismatch between internal representations of the deceased and external reality, which can disrupt emotion regulation and intensify grief. As bereaved individuals repeatedly confront the physical absence of the deceased, they may gradually transform this internal representation, establishing a more abstract internal connection that aligns with external reality. However, in some cases, this transformation remains insecure, and proximity-seeking behaviors persist [3, 6].

Building on this framework, contemporary attachment models conceptualize grief as a dynamic regulatory process involving the ongoing negotiation of proximity-seeking, emotional security, and loss-related representations. Recent work highlights how individual differences in attachment-related processes shape emotional responses and coping trajectories following separation and bereavement. For example, Leroy et al. emphasize the reorganization of the attachment hierarchy after loss, suggesting that continued reward-based searching for the deceased may interfere with emotional regulation and adjustment in some individuals [7]. Similarly, Mikulincer and Shaver frame grief as an attachment-related regulatory process in which continuing bonds, emotion regulation strategies, and defensive processes interact over time to influence post-loss adaptation [8]. Taken together, attachment-based perspectives conceptualize grief as a regulatory process in which proximity-seeking, emotion regulation, and the reorganization of internal representations of the deceased unfold over time, potentially shaping how bereaved individuals engage with loss-related objects.

Broader bereavement models conceptualize grief as a dynamic and multidimensional process of regulation and adaptation following loss. The Dual Process Model (DPM) [9] and the Two-Track Model (TTM) [10, 11] highlight different mechanisms within this process while both emphasizing engagement with the loss and the continuing bond with the deceased. The DPM focuses on oscillation between loss-oriented and restoration-oriented coping, whereas the TTM emphasizes the interaction between biopsychosocial functioning and the ongoing relationship with the deceased. Together, these models suggest that bereavement involves not only alleviating distress but also reorganizing the bond with the deceased in ways compatible with external reality. Recent research has also emphasized the role of meaning-making processes in bereavement, suggesting that difficulties in integrating the loss into one’s broader belief system may contribute to greater grief severity [12]. These perspectives indicate that, beyond emotion regulation and attachment processes, meaning construction plays a central role in shaping grief responses.

The regulation of separation and adaptation to loss has been described as unfolding through multiple processes over time. Within these regulatory processes, some individuals rely not only on internal representations but also on concrete, external supports to manage separation and emotional distress. Within this broader process, certain experiences, such as engagement with loss-related objects (i.e., emotionally significant belongings of the deceased), occupy a gray area in grieving. Winnicott introduced transitional objects as items chosen by children striving for autonomy and separation from their caregivers, creating a transitional space between their inner world and external reality [13]. These objects, which children invest with emotional significance, help maintain a sense of connection to their caregiver while also gaining some autonomy. Volkan and Zintl [14] introduced the concept of linking objects in bereavement, explicitly referring to Winnicott’s notion of transitional objects, while distinguishing it as a grief-specific phenomenon in adulthood. Similarly, Goldstein et al. [15] used the term “transitional objects” to describe meaningful belongings of the deceased that are retained and interacted with after loss. Specifically, these objects are conceptualized as emotionally significant items that bereaved individuals keep and actively engage with, such as visiting, touching, or interacting with them, as part of the grieving process.

The phenomenology of these objects in adult bereavement appears broader and more variable than the classical developmental notion of transitional objects. As also reflected in Volkan’s [14] and Goldstein et al.’s [15] work, engagement with such objects may involve not only comfort and symbolic connection, but also distress, longing, and repeated behavioral interaction. In line with this broader understanding, the present study adopts the more neutral and descriptive term “loss-related objects” to capture the range of emotionally significant belongings of the deceased and the diverse ways in which individuals relate to them. Building on these considerations, loss-related objects may reflect attempts to regulate separation distress through external means, which in some cases may support adaptation, but in others may be associated with heightened emotional distress or difficulties in reorganizing the relationship with the deceased. Importantly, the present study does not assume that these objects serve the same developmental function as transitional objects in Winnicott’s sense (i.e., promoting autonomy or separation), but rather focuses on their behavioral and emotional correlates in adult bereavement.

According to Volkan and Zintl, [14] such objects reflect efforts to safely recalibrate the lost relationship in the external world. They emphasized the distinction between keepsakes or mementos and more intensely engaged loss-related objects, which may become imbued with strong emotional significance, leading bereaved individuals to feel compelled to protect, visit, or treat them with special care. For bereaved individuals, loss-related objects represent and reenact conflicts related to the loss and the impact of death. According to this view, they thus externalize grief, potentially creating an illusion of control over the lost relationship [14]. Building on Winnicott’s formulation of transitional objects as mediators between inner and external reality, Volkan argued that, in adult bereavement, such externally anchored engagements may hinder the reorganization of the internal bond when they become rigid or overinvested [14].

At the same time, loss-related objects differ from the concept of continuing bonds widely discussed in grief literature. Continuing bonds refer to the internal and symbolic continuation of the relationship with the deceased, maintained through memories, meanings, and emotional representations in a flexible and adaptive manner. In contrast, loss-related objects involve the externalization of this relationship onto a concrete item, often marked by intense emotional investment and repetitive or ritualized engagement. Importantly, not all concrete forms of maintaining a bond with the deceased qualify as loss-related objects; for instance, corresponding with the deceased may represent a continuing bond without functioning as a loss-related object or “touchstone.” Loss-related objects more specifically refer to items to which the mourner may cling and repeatedly return. These engagements are often ritualistic or ambivalent and may reflect attempts to recreate a sense of proximity or symbolic connection. Thus, while continuing bonds reflect an internal reorganization of the relationship, loss-related objects may represent its external and sometimes rigid embodiment, and the two should not be conflated. From this perspective, examining interactions with loss-related objects may be valuable, particularly when focusing on the lived and clinically observable aspects of the phenomenon rather than on strict conceptual distinctions from continuing bonds. Rather than drawing a strict categorical distinction, the present study adopts a dimensional approach, focusing on the degree and nature of engagement with loss-related objects.

The limited research conducted on loss-related objects has left unanswered questions about whether they are a universal experience among bereaved individuals. Furthermore, there is limited research on whether the use of objects during mourning is adaptive or maladaptive. For instance, Goldstein et al., using questionnaire-based self-report data collected from bereaved mothers 2–36 months after infant loss, found that 98% of mothers reported keeping objects, and that mothers diagnosed with PGD experienced greater distress when connecting with these objects, although this distress was largely described as restorative [15]. LeDuff et al. reported that engagement with such object**s** was perceived as helpful in mothers’ adaptation to perinatal loss, based on maternal self-reports and clinical observations rather than systematic measures of grief-related outcomes [16]. In addition, many studies have shown that perseverative seeking of the deceased’s physical presence and maintaining bonds with the deceased through concrete objects, such as personal belongings or clothing, are associated with increased PG symptoms [17–20]

### Aims and scope of the study

Despite theoretical explanations regarding the role of loss-related objects in the grieving process, empirical research remains limited. Existing studies have primarily focused on specific loss contexts, such as perinatal or infant loss, and have relied heavily on qualitative reports or clinical observations. Consequently, little is known about the prevalence, phenomenology, and psychological correlations of loss-related object engagement within the general bereaved population.

The present study addresses this gap in three ways. First, it examines the nature and frequency of loss-related objects and their associations with grief symptom severity in a general population sample. Second, it tests whether behavioral and emotional engagement with these objects predict grief severity beyond loss-related variables, attachment, and emotion regulation difficulties. In line with these aims, the study tested two primary hypotheses, presented below.

- H1. Individuals who keep a loss-related object will report higher grief symptom severity than those who do not keep a loss-related object.
- H2. Object-related behavioral interactions (visiting frequency, touching, hugging, and smelling) and emotional engagement (yearning, pain, and discomfort) will be positively associated with grief symptom severity, beyond loss-related characteristics, attachment styles, and difficulties in emotion-regulation.

## Method

### Participants

This study included 250 bereaved individuals, consisting of 204 women (81.6%) and 46 men (18.4%). Participants' age ranged from 18 to 60 years, with a mean age of 26.24 (SD = 9.26). See Table 1 for sample characteristics. Inclusion criteria were being over 18 years old and having experienced the death of a close other. Out of 341 participants, 255 completed all questionnaires in full. In addition, five participants with extreme values on PG-13 were excluded from the analyses. Consequently, the final analyses were conducted with 250 participants.

**Table 1:** Demographic and loss-related variables

	**n**	**%**
Gender		
Female	204	81.6%
Male	46	18.4%
Education Attainment		
Postgraduate	21	8.4%
University graduate	41	16.5%
University students	167	67.1%
High school or below	20	8%
SES^a^		
High	68	27.6%
Middle	142	57.7%
Low	36	14.6%
Relationship with the deceased		
Mother	37	14.9%
Father	32	12.9%
Sibling	14	5.6%
Partner	4	1.6%
Children	2	.08%
Grandparents	83	33.3%
Second-degree relatives^b^	44	17.7%
Friends	33	13.3%
Cause of Death		
Sudden illness	108	43.2%
Expected illness and aging	92	36.8%
Accidents, disasters, or human-made causes	34	13.6%
Suicide	16	6.4%
	M	SD
Age of Bereaved (year)	26.24	9.26
Time since loss (month)	46.85	44.63
Perceived suddenness of the loss (1 = Not at all, 3 = Extremely)	2.32	.71
Perceived violence of the loss (1 = Not at all, 3 = Extremely)	2.00	.70

^a^Socioeconomic status (SES) was measured categorically as low, middle, or high SES

^b^Second-degree relatives: uncles/aunts, nephews/nieces

### Procedure

The study was conducted in accordance with the ethical standards of the Istanbul Medipol University Ethics Committee (Approval No: 179). All participants provided informed consent, and the study was conducted in accordance with the Declaration of Helsinki. Fourth-year psychology students received extra academic course credit for completing the online survey themselves if they had experienced a loss, and for inviting others they knew who had experienced a loss to participate. Although these senior students were familiar with research procedures and the sensitivity of grief and bereavement processes, the researcher provided a detailed briefing on ethical principles and appropriate sensitivity prior to data collection. Before completing the surveys, participants were presented with a consent form that provided information about the study’s participation criteria, the purpose of the study, and the voluntary nature of participation. Individuals who met the participation criteria and voluntarily agreed to participate were asked to complete the scales.

### Measurements

#### Demographic form

A 14-item questionnaire was used to collect sociodemographic information, including age, gender, socioeconomic status (SES), and education level, as presented in Table 1. In addition, loss-related variables were assessed, including age of the deceased, date of death, cause of death, and perceived levels of suddenness and violence of the loss.

#### Loss-related object items

This form, developed based on the study by Goldstein et al., is designed to measure interactions with the loss-related object and the associated emotional experiences [15]. It consists of two categories:

##### Behavioral interactions with the object

Behavioral interaction with the object refers to the frequency of visits and the behavioral interactions with the object (touching, hugging and smelling). This category first evaluates the frequency of visits on a 6-point Likert scale (1 = *Never*, 6 = *Almost every day*). The frequency of behaviors during interactions with the object, including touching, hugging, and smelling the object, is assessed using a 5-point Likert scale ranging from 1 = *Never* to 5 = *Always* (e.g., *When I visit the object, I smell it*). Higher scores indicate more frequent behavioral interactions with the object. Items were not computed into a composite score; each item was analyzed individually. However, a reliability analysis was conducted for the three items together. The Cronbach’s alpha was 0.79. Further reliability analyses are presented in Table 4.

##### Emotional engagement with the object

This category assesses the intensity of emotions experienced during interaction with the loss-related object. Negative emotions included three items assessing discomfort, yearning, and pain (e.g., “When I visit the object, I feel pain”), whereas positive emotions included two items assessing comfort and happiness (e.g., “When I visit the object, I feel happy”). All items were rated on a 5-point Likert scale ranging from 1 (Never) to 5 (Always), with higher scores indicating more frequent emotional experiences. Emotion items were not combined into composite scores and were analyzed individually; nevertheless, reliability analyses were conducted. Cronbach’s alpha was 0.78 for the two positive items and 0.60 for the three negative emotion items. Further reliability analyses are presented in Table 4.

To reduce analytic redundancy and Type-I error risk, analyses focused on core grief-related negative emotions. Yearning, pain, and discomfort were prioritized due to their relevance to separation distress and attachment-related processes. Positive emotions were examined exploratorily and are reported in Appendix Table A1.

#### Experiences in Close Relationships-Revised Short Form (ECR-SF)

The ECR-SF developed by Wei et al. (2007) was used to assess attachment experiences in close relationships [21]. The ECR-SF consists of 12 items rated on a 5-point Likert scale ranging from 1 = *Strongly disagree* to 5 = *Strongly agree* and includes two subdimensions: anxious attachment and avoidant attachment. Higher scores on each dimension indicate higher levels of anxious or avoidant attachment, respectively. The Turkish validity and reliability studies of the ECR-SF were conducted by Savcı and Aysan [22]. Cronbach’s alpha coefficients of 0.90 were reported for both dimensions in the Turkish validation study, whereas internal consistency coefficients in the present sample ranged between 0.63 and 0.65.

#### Difficulties in Emotion Regulation Scale-Brief Form (DERS-16)

DERS-16 was developed by Bjureberg et al. (2016) to assess difficulties in emotion regulation [23]. The scale consists of 16 items and five subdimensions: lack of emotional clarity (*clarity*), difficulties engaging in goal-directed behavior (*goals*), impulse control difficulties (*impulse*), limited access to emotion regulation strategies (*strategies*), and nonacceptance of emotional responses (*nonacceptance*). Items are rated on a 5-point Likert scale ranging from 1 = *Almost never* to 5 = *Almost always*, with higher scores indicating greater difficulties in emotion regulation. The Turkish adaptation of the scale was conducted by Yiğit and Yiğit, with an internal consistency coefficient of 0.92, and subscale coefficients ranging from 0.78 to 0.87 [24]. In the present sample, Cronbach’s alpha internal consistency coefficients ranged between 0.80 and 0.87.

#### Prolonged Grief Scale-13 (PG-13)

PG-13 was developed by Prigerson et al., to assess symptom severity of prolonged grief disorder [25]. The PG-13 consists of a total of 13 items, including 11 items assessing prolonged grief symptoms using a 5-point Likert scale (1 = *Not at all* to 5 = *Several times a day*/*Overwhelmingly*) and two yes/no items assessing the duration of grief and its impact on functioning. The two yes/no items assessing symptom intensity and functional impairment were used to describe diagnostic criteria but were not included in the continuous total score. Higher scores indicate more intense grief symptoms. It is noteworthy that pain, which is among the symptoms of PGD, is not explicitly indicated in the PG-13, which was used in this study, but is included in PG-13-R. The Turkish adaptation of PG-13 was conducted by Işıklı et al. [26]. The Cronbach's alpha internal consistency coefficient of the scale was found to be 0.90. In the present sample, Cronbach’s alpha was 0.89. Because some participants had experienced relatively recent losses, time since loss was included as a control variable in the regression analyses. Therefore, PG-13 scores were interpreted as grief severity rather than as a diagnostic indicator of prolonged grief disorder.

### Data analysis

All statistical analyses were conducted using IBM SPSS Statistics version 27. After summarizing the descriptive statistics related to demographic information and loss-related information in Table 1, findings related to object retention were first presented with descriptive statistics. Subsequently, H1 was tested using independent samples t-tests comparing individuals who keep an object and those who do not on attachment styles, difficulties in emotion regulation, and PG-13. Based on analyses conducted among object keepers, loss-related object items are first presented with their means, standard deviations, skewness, and kurtosis values in Table 4. Subsequently, H2 was tested using a four-step hierarchical multiple regression analysis. Control variables were entered in Step 1, loss-related variables in Step 2, attachment styles and difficulties in emotion regulation in Step 3, and object-related behaviors and emotions in the final step.

## Results

Results are presented as descriptive findings, group comparisons, and regression analyses.

### Sample characteristics and descriptive statistics

Descriptive analysis was performed to identify the demographic and loss characteristics of the sample (see Table 1).

### Findings related to object retention

#### Descriptive statistics by object retention

**Fig. 1:**
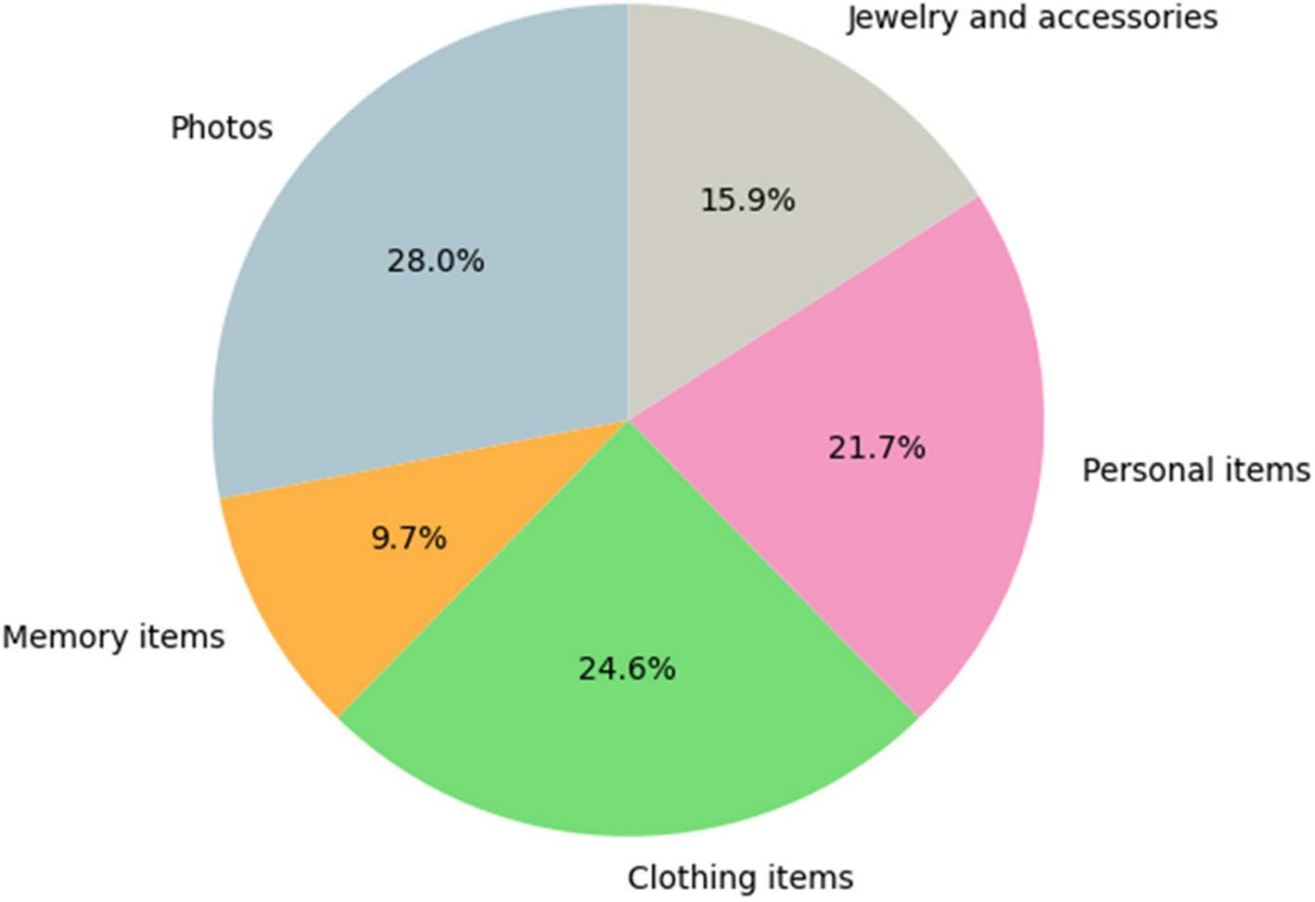
Distribution of kept object types. Note. Percentages indicate the proportion keeping each object type. ^a^Memory items: Gifts from deceased loved ones, such as letters, toys. ^b^Personal items: Phone, comb, hair clips, radio, mug etc.

Descriptive analysis was also conducted to explore the characteristics of objects kept after the loss of a loved one. While 208 (83.2%) of the sample kept objects, 42 participants (16.8%) did not. For these 208 individuals, the most frequently kept items were presented in Table 2 and Fig. 1. Among first-degree family members including mother, father, sibling, partner, and child (*n* = 79), the sub-analysis showed that 88.8% kept objects after their loss, indicating a slight difference in frequency.

**Table 2:** Frequency of kept objects

Kept Objects	n	%
Yes	208	83.2
No	42	16.8

**Kept Object Type**	**n**	**%**
Photos	58	23.2%
Memory items^a^	20	8%
Clothing items	51	20.4%
Personal items^b^	45	18%
Jewelry and accessories	33	13.2%

^a^Memory items: Gifts from deceased loved ones, such as letters, toys

^b^Personal items: Phone, comb, hair clips, radio, mug etc.

#### Group comparisons by object retention

To test H1, participants who kept versus did not keep objects after a loss were compared using independent samples t-tests on attachment styles, difficulties in emotion regulation, and grief symptom severity. Levene’s test indicated that the homogeneity of variance assumption was met for all variables (*p* > 0.05), except ER impulse (*p* < 0.05); therefore, Welch’s t-test was used for DERS-16 impulse. Results showed that participants who kept a loss-related object reported significantly higher PG-13 scores (*t* = 3.36, *p* = 0.001) and greater impulse control difficulties (*t* = 2.07, *p* = 0.009), whereas those who did not keep an object showed higher avoidant attachment (*t* = −2.02, *p* = 0.043). No significant group differences were found for anxious attachment, lack of emotional clarity, difficulties engaging in goal-directed behavior, limited access to emotion regulation strategies, or nonacceptance of emotional responses (all *ps* > 0.05). These findings suggest that those who kept objects tended to report higher grief symptoms and impulse difficulties, whereas those who did not keep objects showed higher avoidant attachment. Results are presented in Table 3.

**Table 3:** Independent samples t-test results

Variable	Keeping (*N* = 208) M (SD)	Not keeping (*N* = 42) M (SD)	t	p	Mean Difference [95% Bootstrap CI]
PG-13	30.60 (9.25)	25.31 (8.64)	3.36	.001	5.28 [2.33, 8.11]
ECR-SF Avoidant	12.14 (3.36)	13.32 (3.59)	−2.02	.043	−1.18 [−2.30, −0.02]
ECR-SF Anxious	18.90 (4.09)	18.37 (4.99)	0.74	.507	0.54 [−1.20, 2.08]
DERS-16 Clarity	4.99 (2.02)	4.73 (2.10)	0.73	.471	0.26 [−0.45, 0.94]
DERS-16 Goals	9.94 (2.96)	9.24 (3.31)	1.34	.208	0.69 [−0.36, 1.80]
DERS-16 Impulse^a^	7.12 (3.30)	6.01 (2.25)	2.76	.007	1.12 [0.32, 1.96]
DERS-16 Strategies	12.96 (4.76)	12.17 (4.84)	0.97	.331	0.79 [−0.81, 2.39]
DERS-16 Nonacceptance	6.63 (3.15)	7.22 (3.60)	−1.06	.333	−0.59 [−1.74, 0.57]

*ECR-SF* Experiences in Close Relationships–Revised Short Form, *DERS-16* Difficulties in Emotion Regulation Scale–Short Form, *PG-13* Prolonged Grief −13 Scale

^a^Welch’s t-test was reported for DERS-16 Impulse

### Findings among loss-related object keepers

#### Item-level descriptive statistics

First, descriptive and item-level statistical indicators for the loss-related object items are presented in Table 4.

**Table 4:** Descriptive statistics and item–total correlations for loss-related object items

Behaviors	M	SD	Min.-Max	Skewness	Kurtosis	Corrected Item-Total Correlation
Visiting Frequency	3.29	1.61	1–6	.52	−1.02	-
Touching	3.51	1.31	1–5	.17	-.79	.56
Hugging	2.30	1.37	1–5	.18	-.82	.68
Smelling	2.22	1.44	1–5	.18	-.81	.66

**Emotions**	**M**	**SD**	**Min.-Max**	**Skewness**	**Kurtosis**	**Corrected Item-Total Correlation**
Feeling Discomfort	1.88	1.15	1–5	1.02	-.05	.32
Feeling Yearning	4.19	1.01	1–5	−1.25	1.06	.40
Feeling Pain	3.37	1.45	1–5	-.33	−1.27	.57

#### Hierarchical regression results

Exploratory correlation analyses examined associations among loss-related object interactions, emotional engagement with the object, PG-13 grief severity scores, and loss-related control variables. Correlation matrices are presented in the Supplementary Materials (Appendix A, Table A1). In addition, correlation coefficients among the study variables are reported in Table A2.

To test H2, a hierarchical multiple regression analysis was conducted to examine associations among object-related behaviors and emotional responses, attachment styles, difficulties in emotion regulation, and grief severity (PG-13). Multicollinearity diagnostics were within acceptable ranges (VIF < 5; tolerance > 0.20). Analyses were based on cases with complete data across all variables (*n* = 143). To address potential Type I error, predictors were entered in theoretically guided blocks. Control variables (suddenness, perceived violence, time since loss, and age of the deceased) were entered at Step 1, followed by attachment styles (Step 2), difficulties in emotion regulation (Step 3), and object-related variables (Step 4). The final model was significant, (*F*(18, 124) = 8.71, *p* < 0.001) and each step contributed additional explained variance in PG-13. Full model fit indices are presented in Table 5.

**Table 5:** Hierarchical regression analysis predicting grief severity (PG-13)

Step 1	Predictors	B	SE	β	t	p	95% CI	Tolerance	VIF
Control Variables	Age of deceased	-.09	.04	-.23	−2.69	.008	[-.16, -.03]	.81	1.23
	Time since loss	-.05	.02	-.25	−3.18	.002	[-.09, -.02]	.93	1.08
	Suddenness	2.34	1.03	.18	2.27	.025	[.30, 4.39]	.88	1.13
	Violence	.88	1.18	.06	.75	.455	[−1.44, 3.20]	.85	1.17
Model 1 fit: R^2^ =.195, Adj. R^2^ =.172, F(4, 138) = 8.36, *p* <.001									

**Step 2**	**Predictors**	**B**	**SE**	**β**	**t**	**p**	**95% CI**	**Tolerance**	**VIF**
Control variables	Age of deceased	-.11	.04	-.26	−3.11	.002	[-.18, -.04]	.79	1.27
	Time since loss	-.05	.02	-.26	−3.28	.001	[-.09, -.02]	.93	1.08
	Suddenness	2.09	1.04	.17	2.01	.046	[.04, 4.15]	.83	1.20
	Violence	.74	1.16	.05	1.98	.050	[.00, 0.86]	.85	1.18
Attachment	Avoidant	.43	.22	.15	1.98	.050	[.00,.86]	.96	1.04
	Anxious	.26	.18	.11	1.43	.154	[-.10,.63]	.94	1.07
Model 2 fit: R^2^ =.231, Adj. R^2^ =.198 ΔR^2^ =.036, ΔF(2, 136) = 3.23, *p* =.043									

Step 3	**Predictors**	**B**	**SE**	**β**	**t**	**p**	**95% CI**	**Tolerance**	**VIF**
Control variables	Age of deceased	-.09	.03	-.22	−2.71	.008	[-.16, -.02]	.76	1.31
	Time since loss	-.05	.02	-.23	−3.14	.002	[-.08, -.02]	.91	1.10
	Suddenness	1.78	.99	.14	1.80	.074	[-.18, 3.73]	.83	1.21
	Violence	.86	1.10	.06	.78	.437	[−1.32, 3.04]	.83	1.20
Attachment	Avoidant	.48	.21	.17	2.32	.022	[.07,.89]	.93	1.07
	Anxious	-.06	.21	-.03	-.29	.775	[-.47,.35]	.66	1.52
Difficulties in Emotion Regulation	Clarity	.24	.37	.05	.63	.531	[-.50,.97]	.72	1.38
	Goal	-.18	.31	-.06	-.59	.559	[-.80,.44]	.50	1.98
	Impulse	.24	.26	.09	.91	.364	[-.28,.75]	.58	1.74
	Strategy	.83	.23	.44	3.58	<.001	[.37, 1.29]	.34	2.96
	Unacceptance	-.69	.28	-.24	−2.49	.014	[−1.24, -.14]	.53	1.89
Model 3 fit: R^2^ =.345, Adj. R^2^ =.290 ΔR^2^ =.113, ΔF(5, 131) = 4.52, *p* =.001									

Step 4	**Predictors**	**B**	**SE**	**β**	**t**	**p**	**95% CI**	**Tolerance**	**VIF**
Control variables	Age of deceased	-.05	.03	-.12	−1.73	.086	[-.11,.01]	70	1.43
	Time since loss	-.03	.01	-.16	−2.31	.022	[-.06, -.01]	.76	1.32
	Suddenness	1.03	.86	.08	1.20	.233	[-.68, 2.74]	.77	1.29
	Violence	.05	.97	.00	.05	.963	[-.87, 1.97]	.77	1.31
Attachment	Avoidant	.37	.18	.13	2.06	.042	[.01,.72]	.89	1.12
	Anxious	-.10	.18	-.04	-.54	.593	[-.46,.26]	.60	1.66
Difficulties in Emotion Regulation	Clarity	.13	.32	.03	.41	.685	[-.50,.76]	.70	1.42
	Goal	-.18	.28	-.06	-.65	.520	[-.73,.37]	.46	2.17
	Impulse	.16	.23	.06	.72	.474	[-.29,.61]	.54	1.87
	Strategy	.48	.22	.25	2.23	.027	[.05,.91]	.28	3.57
	Unacceptance	-.25	.25	-.09	−1.00	.318	[-.75,.25]	.73	1.37
Loss Related Object Items	Visiting frequency	.88	.44	.14	2.00	.048	[.01, 1.75]	.73	1.37
	Touching	.38	.54	.05	.70	.488	[-.69, 1.44]	.63	1.59
	Hugging	-.23	.58	-.03	-.40	.694	[−1.38,.92]	.48	2.10
	Smelling	1.35	.56	.21	2.39	.018	[.23, 2.46]	.46	2.16
	Feeling Discomfort	.55	.62	.07	.90	.370	[-.66, 1.77]	.61	1.64
	Feeling Yearning	1.12	.75	.11	1.49	.139	[-.37, 2.61]	.63	1.58
	Feeling Pain	1.73	.52	.27	3.34	.001	[.70, 2.76]	.53	1.88
Model 4 fit: R^2^ =.558, Adj. R^2^ =.494 ΔR^2^ =.214, ΔF(7, 124) = 8.58, *p* <.001									

In the hierarchical regression analysis, age of the deceased (*β* = −0.23, *p* = 0.008), time since loss (*β* = −0.25, *p* = 0.002), and suddenness of the death (*β* = 0.18, p = 0.025) were included as control variables and were significantly associated with grief severity in the initial model. After adding attachment variables, age of the deceased (*β* = −0.26, *p* = 0.002) and time since loss (*β* = −0.26, *p* = 0.001) remained significant, while avoidant (*β* = 0.15, *p* = 0.050) and anxious attachment (*β* = 0.11, *p* = 0.154) were not significant predictors. With the inclusion of emotion regulation variables, avoidant attachment (*β* = 0.17, *p* = 0.022) became significant. Difficulties in emotion regulation strategies showed a strong positive association (*β* = 0.44, *p* < 0.001), while non-acceptance was negatively associated (*β* = −0.24, *p* = 0.014). In the final model, time since loss (*β* = −0.16, *p* = 0.022), avoidant attachment (*β* = 0.13, *p* = 0.042), and difficulties in emotion regulation strategies (*β* = 0.25, *p* = 0.027) remained significant. Visiting frequency (*β* = 0.14, *p* = 0.048), smelling the object (*β* = 0.21, *p* = 0.018), and experiencing pain during interaction (*β* = 0.27, *p* = 0.001) were also significantly associated with higher grief severity. Overall, grief symptoms were associated with avoidant attachment, difficulties in emotion regulation strategies, and specific object-related experiences, particularly those involving sensory engagement and emotional pain.

## Discussion

This study examined the role of objects kept after a loss in grief symptoms. Specifically, we compared individuals who kept objects with those who did not and tested whether behavioral and emotional engagement with these objects predicted grief severity beyond attachment styles, emotion regulation difficulties, and loss-related factors.

Descriptive findings indicated that 83.2% of participants retained loss-related objects, including photos, memory items, clothing, personal belongings, and jewelry. These objects were primarily associated with emotional attachment to the deceased. The prevalence of kept objects in this study is consistent with the findings of Goldstein et al. who reported that most bereaved mothers kept objects after loss [15]. Similarly, Gibson [27] found that keepsakes, such as photographs and clothing, were preserved by individuals who had experienced the death of a family member. These objects likely serve as emotional bridges, facilitating the bereaved individual’s adjustment to the loss by maintaining a connection to the deceased. This aligns with Volkan's concept of "linking phenomena," whereby objects can create the illusion of continued contact with the lost person, aiding in emotional adjustment during grief [28].

In line with the first aim of the study, the initial finding indicated that individuals who retained objects reported higher levels of grief symptoms. This finding is consistent with Goldstein et al. [15] who noted that nearly half of the individuals who kept objects met the criteria for prolonged grief disorder. However, it is important to note that the present study measures prolonged grief using PG-13 scores rather than a diagnostic algorithm; therefore, the findings suggest a correlation rather than a definitive diagnosis of prolonged grief. These results indicate that individuals who retain objects may experience more intense emotional responses during the grieving process. It is also worth noting that the sample of bereaved mothers studied by Goldstein et al. overall exhibited notably elevated rates of PGD symptoms compared with other kindred groups [29]. Another difference observed within the scope of the first aim was that participants who did not keep objects reported higher avoidant attachment scores, consistent with previous research. Studies have shown that individuals with avoidant attachment tend to suppress emotional bonds and distance themselves from close relationships during grief [9, 30]. For these individuals, disengaging from loss-related objects after loss may reflect a broader strategy of minimizing emotional vulnerability [31]. They may engage less with the grief process, potentially suppressing emotions related to the loss. In contrast, no significant difference was found between individuals who retained an object and those who did not in terms of attachment anxiety. This finding suggests that the mere act of keeping a belonging may not, in itself, be related to anxious attachment. However, examining attachment in relation to the quality of individuals’ relationships with the object may provide further insight.

In addition to attachment-related processes, the findings highlight the relevance of emotion regulation in understanding object retention. Individuals who retained loss-related objects exhibited higher impulse-control difficulties, suggesting that those who struggle to inhibit behavioral responses under distress may be more likely to retain such objects. Retaining a loss-related object may thus represent a concrete behavioral response under heightened emotional arousal. Difficulties in impulse control further intensify emotional responses during interactions with the objects that symbolize the loss, particularly when adaptive emotion regulation strategies are limited [32]. Importantly, such emotional responses may not be specific to grief-related processes alone, but may instead reflect broader difficulties in emotion regulation, given that impulse control is implicated across multiple domains of emotional functioning. Anecdotally, however, kept objects are seen by their keepers as adaptive and precious, at least in some populations [33].

In line with the second aim of the study, behavioral and emotional experiences during contact with loss-related objects were associated with grief symptom severity, beyond the contributions of attachment styles and difficulties in emotion regulation, while controlling for loss-related variables. In the final model, time since loss emerged as a significant predictor, with shorter time since loss associated with higher grief symptom severity. This pattern likely reflects ongoing adjustment processes, rather than a direct causal effect of time itself. Accordingly, time since loss is interpreted as a proxy for broader adaptation processes. This finding is consistent with prior research suggesting that grief intensity tends to decrease over time as individuals gradually adjust to the loss.

The perceived suddenness of the death contributed to grief severity at earlier stages of the model, supporting literature linking unexpected loss to heightened emotional disruption, adjustment difficulties, and increased prolonged grief symptoms [34, 35]. Sudden deaths may abruptly shatter assumptive worlds and hinder meaning-making processes, thereby increasing vulnerability to prolonged grief [36]. Contrary to expectations, violent death did not uniquely predict prolonged grief severity in the final model. This may suggest that perceived suddenness, rather than violence, plays a more central role in shaping grief responses. Suddenness may more directly disrupt meaning-making and emotional adjustment [37]. Descriptive findings showing higher suddenness and limited variability in perceived violence may also explain its reduced predictive contribution. Also, the age of the deceased remained significant until the final step of the model, with younger deaths often related to more unexpected and traumatic causes, leading to more intense grief symptoms [38, 39]. However, when psychological variables and behavioral and emotional engagement with loss-related objects are included in the model, they appear to be associated with processes related to grief severity, thereby reducing the unique contribution of perceived suddenness and age of the deceased.

When psychological variables were introduced, avoidant attachment (with a small coefficient) and difficulties in accessing emotion regulation strategies remained significant predictors until the final step of the model. Avoidant attachment is characterized by emotional suppression, interpersonal distancing, and cognitive avoidance, which may hinder integration of the loss and contribute to the persistence of symptoms. Rooted in early relational experiences, attachment styles shape responses to separation and loss [40]. Consistent with this framework, prior research has also linked avoidant attachment to prolonged grief symptoms [19, 41].

Although earlier findings showed that individuals who did not retain objects reported higher avoidant attachment, this pattern may appear contradictory, yet it may reflect processes operating at different levels. Avoidant attachment is generally associated with strategies aimed at maintaining emotional distance; however, these strategies may not always be effective and, in some cases, may be linked to greater grief severity. In this context, engagement with objects may be related to these processes; however, what appears to be more critical is not the presence of the object itself, but the individual’s way of regulating emotions.

Among emotion regulation dimensions, Unacceptance was significant in earlier steps of the model but did not retain significance in the final step when object-related variables were included. Although unacceptance reflects a tendency to reject or struggle with emotional responses, it may not be a strong mechanism in the context of bereavement. Grief involves not only the acceptance of negative emotions but also the ability to regulate emotional responses; in line with this, difficulties in accessing regulation strategies showed the strongest association with grief severity, highlighting the central role of regulatory capacity in bereavement adaptation. Consistent with prior research, difficulties in accessing effective regulation strategies have been repeatedly linked to grief and prolonged grief symptoms [42, 43]. This may more directly reflect how individuals respond to and cope with loss-related emotional experiences. More broadly, difficulty in regulating emotions is linked to maladaptive emotional responses, emphasizing the need for effective emotion regulation strategies to facilitate grief adaptation [44].

Finally, the frequency of object visits, smelling the object, and feelings of pain remained associated with grief severity even after controlling for loss-related variables, as well as attachment styles and difficulties in emotion regulation. The positive association between visiting frequency and PG-13 scores may indicate that frequent contact with the object is linked to heightened grief-related distress. Visiting the object may, in turn, be associated with the persistence of grief symptoms. Alternatively, more frequent visits may reflect greater underlying grief severity rather than directly contribute to it. Among these variables, emotional pain appeared particularly prominent. Pain experienced during interactions with the object was strongly associated with grief symptoms, whereas discomfort and yearning did not show significant associations. This association may partly reflect conceptual overlap between pain-related experiences and grief symptom measures. While pain is specified in the PG-13-R, it is not directly included in the PG-13 used in this study. Nevertheless, pain was strongly associated with grief severity. Prior work conceptualizing yearning as involving intense emotional suffering highlights the potential role of emotional pain in the grieving process, particularly in prolonged grief [26]. Accordingly, the present findings suggest that pain may be one component of grief severity, underscoring the potential value of assessing emotional pain in bereavement interventions.

In addition to visiting frequency and emotional pain, smelling the object was uniquely associated with grief symptom severity. This association may reflect the close links between olfaction and emotion-related neural systems [45, 46]. Accordingly, sensory experiences such as smell may be linked to stronger emotional attachment to the deceased and more severe grief-related symptoms [47]. Within the limbic system hypothesis [48] these findings suggest that olfactory cues may intensify emotional responding through limbic pathways; however, this interpretation remains tentative and should be examined in future studies using controlled olfactory stimulation paradigms. In contrast, tactile contact (touching or hugging the object) was not significantly associated with grief severity in the regression analyses. However, as shown in Appendix A1, tactile interactions were significantly correlated with grief symptom severity (PG-13) at the bivariate level. This suggests that while tactile contact may relate to grief severity in simple associations, its contribution does not remain significant when controlling for other psychological and loss-related variables.

### Limitations

The study has several limitations. The relatively small sample size and the high proportion of university students may limit the generalizability of the findings. In addition, most losses were due to natural causes and involved less central relationships, which may restrict applicability to more intimate losses. Participant characteristics, such as age, kinship to the deceased, life stage, and relationship quality (e.g., closeness, conflict, ambivalence), may further constrain interpretation. Attachment was assessed using a general measure (ECR), and many participants had not lost a primary attachment figure, which may also limit interpretation.

Emotional responses were assessed using a limited set of negative affective states, reflecting a distress-focused approach that may have increased sensitivity to negative outcomes while underrepresenting positive emotional experiences. Although positive responses were assessed, they were not the primary focus and are reported in Appendix A. Object-related behaviors were also analyzed separately rather than as a composite, which may limit capturing their shared variance.

The cross-sectional design precludes causal inferences. In addition, object characteristics were not examined in detail, and key loss-related variables (e.g., cause of death, kinship) were not systematically analyzed. Finally, unequal group sizes should be considered when interpreting group comparisons, as this imbalance may have influenced the results.

### Clinical implications

These findings have practical implications for clinical assessment and intervention in bereavement. Clinicians may benefit from assessing individuals’ engagement with loss-related objects, particularly when such interactions are frequent, emotionally intense, or associated with distress. These patterns may signal difficulties in regulating separation distress or reorganizing the relationship with the deceased. Rather than focusing on the mere presence of such objects as adaptive or maladaptive, it may be more informative to examine how individuals relate to them.

Loss-related objects may provide clinically relevant insight into how individuals regulate proximity, emotional distress, and ongoing bonds with the deceased. In particular, the emotional responses experienced during interactions with these objects can offer valuable information about ongoing grief processes. Exploring the presence, meaning, and emotional impact of these objects may therefore enrich clinical assessment, especially when identifying individuals at risk for more persistent grief responses.

From an intervention perspective, loss-related objects may also offer a practical entry point in therapy, particularly when individuals struggle to articulate their grief experiences. Discussing tangible objects associated with the deceased may facilitate the exploration of emotional pain in a clinically sensitive manner. In this sense, such objects may function not only as symbols of loss but also as observable and clinically useful indicators that can support both assessment and intervention.

## Conclusion

This study underscores the role of loss-related objects in grief symptom severity beyond loss related characteristics, attachment styles, and difficulties in emotion regulation. Individuals who retain such objects tended to report higher grief severity, lower avoidant attachment, and greater impulse control difficulties. Object related factors, including visiting frequency, sensory engagement such as smelling, and emotional responses, particularly pain, were also associated with grief severity. These findings suggest that the psychological significance of loss-related objects lies not merely in their presence but in how individuals engage with them. Overall, the results point to potential pathways linking attachment and emotion regulation to emotional intensity during interactions with retained objects, highlighting the need for longitudinal and mediation based research.

## Supplementary Information

Supplementary Material 1.

## Data Availability

The datasets used and analysed during the current study are available from the corresponding author on reasonable request.
