# Supplementary material for: Behavioral interactions and emotional engagement with loss-related objects in bereavement: associations with grief symptom severity
Source: BMC Psychol. 2026 May 21;14:1042. doi: 10.1186/s40359-026-04784-z (PMC13366898; doi:10.1186/s40359-026-04784-z)
Supplement: Supplementary file 1 — Supplementary Material 1. [file 40359_2026_4784_MOESM1_ESM.docx]

**Appendix A**

Table A1.

*Exploratory Correlations with loss object related items and PG-13*

| **Loss object-related items** | **PG-13** | **Age** | **Age of deceased** | **Time since loss** | **Suddenness of death** | **Perceived violence of death** |
| --- | --- | --- | --- | --- | --- | --- |
| **Behaviors** | | | | | | |
| Visiting frequency | .36** | .10 | -.02 | -.27** | .09 | .01 |
| Touching | .24** | -.04 | -.02 | .07 | -.01 | .02 |
| Hugging | .37** | .06 | .03 | -.08 | .05 | .10 |
| Smelling | .42** | .12 | -.02 | -.09 | -.02 | -.02 |
| **Negative Emotions** | | | | | | |
| Discomfort | .29** | -.15 | -.19* | -.06 | .22** | .14 |
| Yearning | .34** | .08 | -.12 | .07 | .06 | .15* |
| Pain | .54** | .14 | -.22** | -.12 | .20** | .19* |
| **Positive Emotions** | | | | | | |
| Relief | -.09 | .11 | .06 | .11 | -.04 | -.10 |
| Happiness | -.17* | .20** | .16* | .09 | -.16* | -.13 |

**p* < .05, ***p* < .001. PG-13 = PG-13 = Prolonged Grief-13 scale.

Table A2.

*Correlations among study variables*

| **Variable** | 1 | 2 | 3 | 4 | 5 | 6 | 7 | 8 | 9 | 10 | 11 | 12 | 13 | 14 | 15 | 16 | 17 | 18 | 19 |
| --- | --- | --- | --- | --- | --- | --- | --- | --- | --- | --- | --- | --- | --- | --- | --- | --- | --- | --- | --- |
| 1. PG-13 | - |  |  |  |  |  |  |  |  |  |  |  |  |  |  |  |  |  |  |
| 2. Avoidant Attachment | .13 | - |  |  |  |  |  |  |  |  |  |  |  |  |  |  |  |  |  |
| 3. Anxious  Attachment | .16* | .01 | - |  |  |  |  |  |  |  |  |  |  |  |  |  |  |  |  |
| 4. DERS-16 Open | .26** | .12 | .31** | - |  |  |  |  |  |  |  |  |  |  |  |  |  |  |  |
| 5. DERS-16 Goal | .21** | -.01 | .40** | .43** | - |  |  |  |  |  |  |  |  |  |  |  |  |  |  |
| 6. DERS-16 impulse | .25** | -.04 | .30** | .39** | .54** | - |  |  |  |  |  |  |  |  |  |  |  |  |  |
| 7. DERS-16 strategy | .33** | .00 | .53** | .54** | .67** | .63** | - |  |  |  |  |  |  |  |  |  |  |  |  |
| 8. DERS-16 Unacceptance | .12 | .05 | .42** | .38** | .43** | .56** | .63** | - |  |  |  |  |  |  |  |  |  |  |  |
| 9. Visiting Frequency | .36** | .04 | .07 | .15* | .03 | .11 | .15* | .02 | -- |  |  |  |  |  |  |  |  |  |  |
| 10. Touching | .24** | -.01 | .17* | .06 | .10 | -.05 | .07 | -.01 | .12 | - |  |  |  |  |  |  |  |  |  |
| 11. Hugging | .37** | .07 | .18* | .11 | .15 | .06 | .15* | .04 | .15* | .51** | - |  |  |  |  |  |  |  |  |
| 12. Smelling | .42** | -.03 | .01 | .16* | .12 | .12 | .17* | -.09 | .19* | .48** | .64** | - |  |  |  |  |  |  |  |
| 13. Discomfort | .29** | .04 | .19* | .29** | .23** | .24** | .40** | .22** | -.11 | .00 | .06 | .00 | - |  |  |  |  |  |  |
| 14. Yearning | .34** | -.03 | .01 | .06 | .09 | -.00 | .06 | -.07 | .12 | .34** | .25** | .25** | .10 | - |  |  |  |  |  |
| 15. Pain | .54** | -.04 | .16* | .14 | .16* | .10 | .22** | .05 | .18* | .25** | .42** | .34** | .37** | .44** |  |  |  |  |  |
| 16. Time Since Loss | -.21** | .03 | -.04 | .02 | -.03 | -.05 | -.04 | -.00 | -.14* | -03 | -.11 | -.11 | -.08 | .09 | -.10 | - |  |  |  |
| 17. Age of Deceased | -.11 | -.07 | -.08 | -.07 | -.06 | -.01 | .01 | .06 | -.12 | -.20** | -.06 | -.07 | -.12 | -.05 | .15* | .10 | - |  |  |
| 18. Suddenness | .17* | .01 | .10 | .02 | .04 | .04 | .10 | -.06 | .10 | -.01 | .05 | -.02 | .22** | .07 | .21** | -.03 | .01 | - |  |
| 19. Violence | .08 | -.03 | .02 | -.00 | .08 | .06 | .04 | .01 | .02 | .04 | .10 | -.02 | .15 | .16 | .20** | .22** | -.08 | .13 | - |

*Note*. **p* < .05, ***p* < .001. DERS-16 = Difficulties in Emotion Regulation Scale–Short Form; PG-13 = Prolonged Grief-13 Scale
